# Non-Bacterial Thrombotic Endocarditis: A Case of Metastatic Pancreatic Cancer Masquerading as Infective Endocarditis

**DOI:** 10.7759/cureus.9103

**Published:** 2020-07-09

**Authors:** Gurchetan Randhawa, Awais Aslam, Maria J Suarez, Yu Shia Lin, Margaret Kuhn-Basti

**Affiliations:** 1 Internal Medicine, Maimonides Medical Center, Brooklyn, USA; 2 Infectious Diseases, Maimonides Medical Center, Brooklyn, USA

**Keywords:** non-bacterial thrombotic endocarditis, pancreatic cancer, infectious endocarditis, echocardiogram

## Abstract

This case illustrates a rare, underdiagnosed disease, with a high mortality rate that is frequently misdiagnosed as acute bacterial endocarditis. Clinicians should include non-bacterial thrombotic endocarditis (NBTE) as a differential diagnosis in patients with culture-negative endocarditis, so that its underlying etiology can be further investigated.

## Introduction

Non-bacterial thrombotic endocarditis (NBTE) is a rare syndrome, with a similar presentation to infective endocarditis (IE) [1-3]. The cause of NBTE is the presence of a hypercoagulable state due to an underlying malignancy, or an autoimmune disease, predisposing to thrombus formation on cardiac valves (most commonly involving the aortic and mitral valves) [1]. An antemortem diagnosis of NBTE is exceedingly rare [4].

## Case presentation

**Figure 1:**
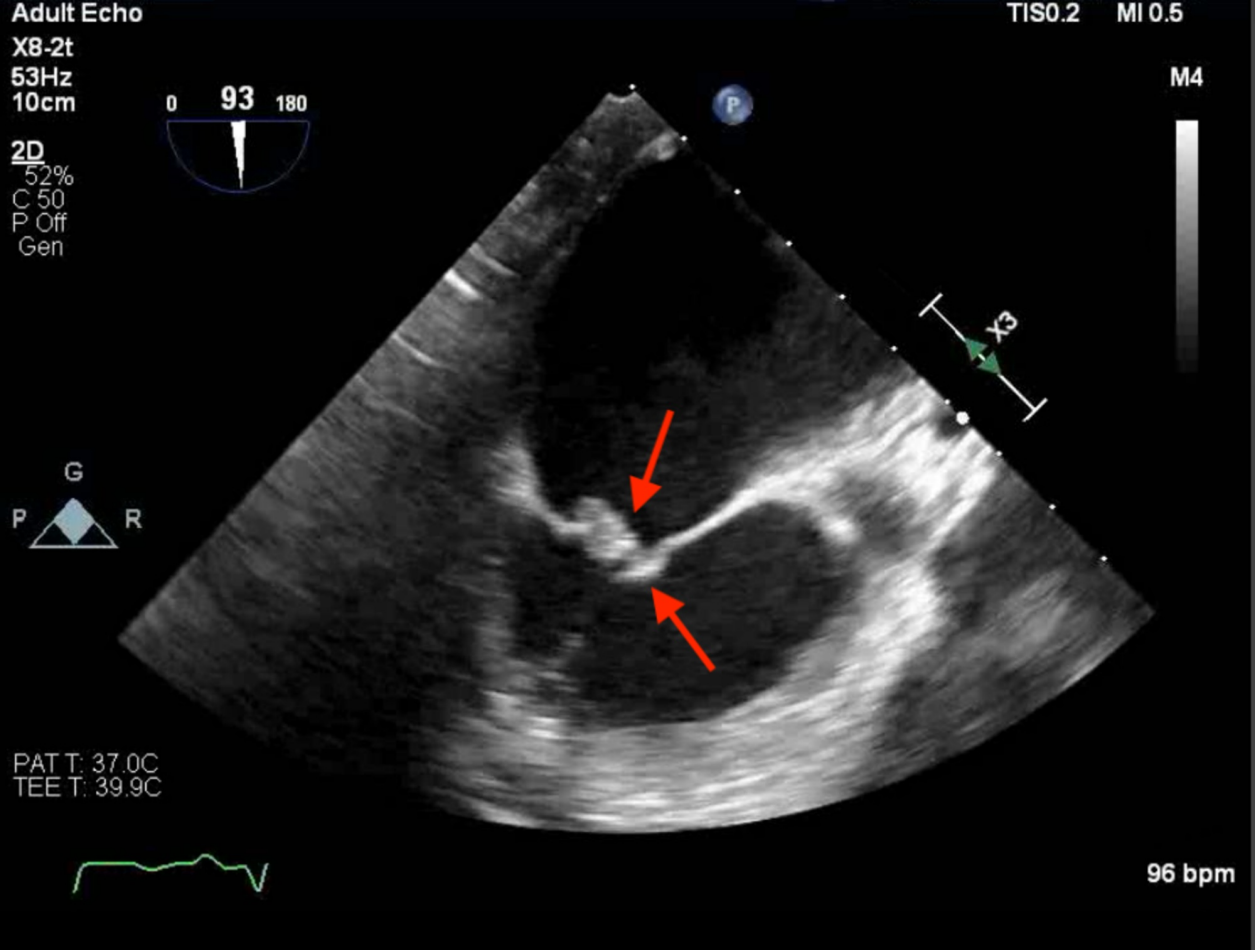
Transesophageal echocardiogram (TEE) The results of a TEE study: echogenic densities, approximately 0.6 cm in size, involving both anterior and posterior mitral leaflets, primarily at the tips, suggestive of endocarditis.

**Figure 2:**
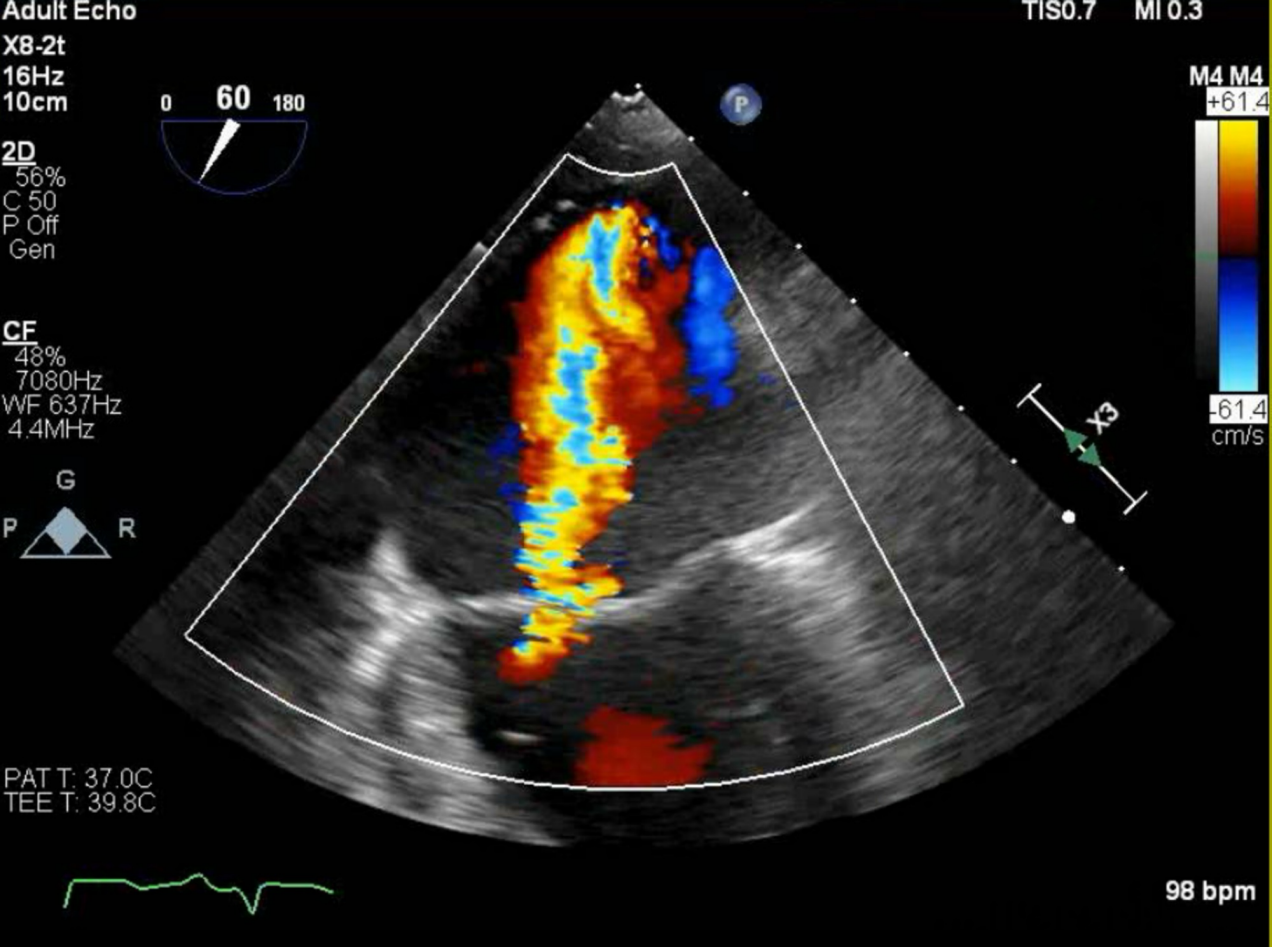
Transesophageal echocardiogram (TEE) This TEE image demonstrates regurgitant flow over the mitral valve.

A 67-year-old male presented to the emergency department for loss of vision, slurred speech, and altered mental status for one day. He experienced symptoms two months prior to admission, including night sweats, a 15-pound weight loss, but denied fever. He is an active smoker and cocaine user. His past medical history includes hypertension, type II diabetes mellitus, chronic kidney disease, and bilateral lower extremity deep venous thrombosis. Upon admission, he presented with the following vital signs: temperature: 101.1˚F; blood pressure: 118/65 mmHg; heart rate: 72 beats/minute; respiratory rate: 15 breaths/minute. On examination, he was confused with anisocoria, and non-reactive pupils. The remainder of his cardiopulmonary examination was normal. His initial laboratory results were significant for leukocytosis of 19.3 K/μL, with an 82% neutrophil count, and a C-reactive protein level of 105 mg/dL. An MRI study of the head indicated bilateral thalamic, left midbrain, pons, and bilateral cerebellar infarcts with a mass effect. A transesophageal echocardiogram (TEE) revealed multiple echogenic densities that were approximately 0.6 cm in size, involving both the anterior, and posterior mitral leaflets (Figures 1, 2). He was started on empiric broad-spectrum antibiotics for suspected IE. Due to a combination of multiple negative blood cultures, persistent fever while on antibiotics, and weight loss, there was a suspicion for NBTE.

**Figure 3:**
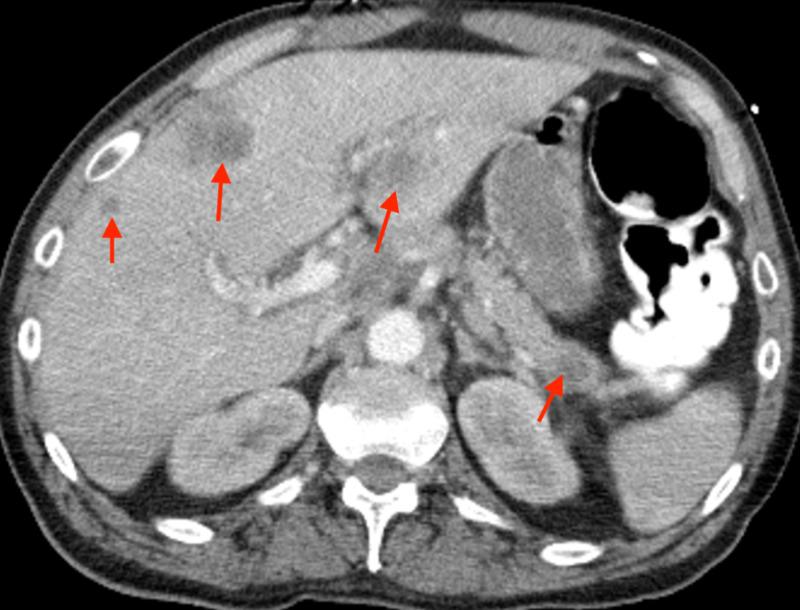
CT of the abdomen with contrast The study reveals diffuse metastatic disease found throughout the liver and a heterogeneous mass with areas of necrosis at the distal body and tail of the pancreas. These findings are consistent with a metastatic pancreatic carcinoma.

CT of the chest, abdomen, and pelvis revealed bilateral pulmonary emboli, with metastatic disease found throughout the liver. The study also revealed a heterogeneous mass, with areas of necrosis at the distal body/tail of the pancreas, consistent with a metastatic pancreatic carcinoma (Figure 3). Antibiotics were discontinued, and unfractionated heparin was initiated. 

## Discussion

The pathophysiology of pancreatic cancer and NBTE involves the release of prothrombotic molecules from cancer cells, such as tissue factor, cancer procoagulant, and mucin, which form a hypercoagulable state [1].

NBTE and IE are difficult to distinguish. However, there are several differences between the two conditions. In NBTE, patients are less likely to have fever and leukocytosis. In addition, the vegetations are smaller than 1 cm and friable. They rarely cause significant hemodynamic instability, valvular destruction, or cardiac murmurs [2]. Recurrent emboli are considered a hallmark feature of NBTE, occurring in up to 50% of patients, which are widely distributed in the body, involving the kidneys, spleen, skin, and extremities [3,5].

Both NBTE and IE are often evaluated with echocardiography. A TEE is more sensitive than a transthoracic echocardiogram; however, a retrospective study indicated that TEE studies were able to detect vegetations in only 18% of NBTE patients [6].

Fever with leukocytosis stemming from embolic complications often makes the diagnosis of NBTE challenging, as in our patient. It is important to rule out IE in order to avoid the overuse of antibiotics and antimicrobial resistance.

Therapy of NBTE involves treating its underlying etiology, and effective systemic anticoagulation, considering 50% of patients with NBTE present with systemic emboli [5]. Unfractionated heparin remains the anticoagulant of choice, as warfarin is implicated in recurrent thromboembolic events, and the efficacy of newer anticoagulant medications remains unknown.

## Conclusions

NBTE is a rare, devastating condition, which is clinically difficult to distinguish from IE. Although the two conditions are difficult to discern, clinicians should be aware of their key differences. Due to its catastrophic complications, antemortem cases of NBTE are very rare. Early diagnosis relies upon a strong clinical suspicion. NBTE should be considered by clinicians as a differential diagnosis in patients with culture negative endocarditis and multiple embolic complications, prompting further investigation of the underlying etiology.

## References

[1] Moţăţăianu A, Maier S, Gothard A, Bajkó Z, Bălaşa R (2018). Severe fatal systemic embolism due to non-bacterial thrombotic endocarditis as the initial manifestation of gastric adenocarcinoma: case report. J Crit Care Med.

[2] Asopa S, Patel A, Khan OA, Sharma R, Ohri SK (2007). Non-bacterial thrombotic endocarditis. Eur J Cardiothorac Surg.

[3] Shibata N, Matsumoto K, Kitamura S, Sakashita A, Kizawa Y, Hirata KI (2018). Nonbacterial thrombotic endocarditis concomitant with repeated systemic embolization that received palliative care based on the antemortem diagnosis. Intern Med.

[4] Piovanelli B, Rovetta R, Bonadei I, Vizzardi E, D'Aloia A, Metra M (2013). Nonbacterial thrombotic endocarditis in pancreatic cancer. Monaldi Arch Chest Dis.

[5] el-Shami K, Griffiths E, Streiff M (2007). Nonbacterial thrombotic endocarditis in cancer patients: pathogenesis, diagnosis, and treatment. Oncologist.

[6] Dutta T, Karas MG, Segal AZ, Kizer JR (2006). Yield of transesophageal echocardiography for nonbacterial thrombotic endocarditis and other cardiac sources of embolism in cancer patients with cerebral ischemia. Am J Cardiol.

